# Pathogenic substitution of IVS15 + 5G > A in *SLC26A4* in patients of Okinawa Islands with enlarged vestibular aqueduct syndrome or Pendred syndrome

**DOI:** 10.1186/1471-2350-14-56

**Published:** 2013-05-24

**Authors:** Akira Ganaha, Tadashi Kaname, Kumiko Yanagi, Kenji Naritomi, Tetsuya Tono, Shin-ichi Usami, Mikio Suzuki

**Affiliations:** 1 Department of Otorhinolaryngology-Head and Neck Surgery, University of the Ryukyus, Okinawa, Japan; 2 Department of Medical Genetics, University of the Ryukyus, Okinawa, Japan; 3 Department of Otorhinolaryngology-Head and Neck Surgery, University of Miyazaki, Miyazaki, Japan; 4 Department of Otorhinolaryngology, Shinshu University School of Medicine, Nagano, Japan

## Abstract

**Background:**

Pendred syndrome (PS) and nonsyndromic hearing loss associated with enlarged vestibular aqueduct (EVA) are caused by *SLC26A4* mutations. The Okinawa Islands are the southwestern-most islands of the Japanese archipelago. And ancestral differences have been reported between people from Okinawa Island and those from the main islands of Japan. To confirm the ethnic variation of the spectrum of *SLC26A4* mutations, we investigated the frequencies of *SLC26A4* mutations and clinical manifestations of patients with EVA or PS living in the Okinawa Islands.

**Methods:**

We examined 22 patients with EVA or PS from 21 unrelated families in Okinawa Islands. The patient’s clinical history, findings of physical and otoscopic examinations, hearing test, and computed tomography (CT) scan of the temporal bones were recorded. To detect mutations, all 21 exons and the exon–intron junctions of *SLC26A4* were sequenced for all subjects. Quantitative reverse-transcription polymerase chain reaction (qRT-PCR) for *SLC26A4* and calculations using the comparative CT (2^−ΔΔCT^) method were used to determine the pathogenicity associated with gene substitutions.

**Results:**

*SLC26A4* mutations were identified in 21 of the 22 patients. We found a compound heterozygous mutation for IVS15 + 5G > A/H723R in nine patients (41%), a homozygous substitution of IVS15 + 5G > A in six patients (27%), and homozygous mutation for H723R in five patients (23%). The most prevalent types of *SLC26A4* alleles were IVS15 + 5G > A and H723R, which both accounted for 15/22 (68%) of the patients. There were no significant correlations between the types of *SLC26A4* mutation and clinical manifestations. Based on qRT-PCR results, expression of *SLC26A4* was not identified in patients with the homozygous substitution of IVS15 + 5G > A.

**Conclusions:**

The substitution of IVS15 + 5G > A in *SLC26A4* was the most common mutation in uniquely found in patients with PS and EVA in Okinawa Islands. This suggested that the spectrum of *SLC26A4* mutation differed from main islands of Japan and other East Asian countries. The substitution of IVS15 + 5G > A leads to a loss of *SLC26A* expression and results in a phenotype of PS and EVA.

## Background

**Figure 1:**
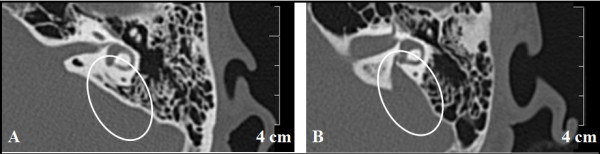
**Computed tomography of the temporal bone showing an enlarged vestibular aqueduct.** Circles show the vestibular aqueduct. The vestibular aqueduct is not identified in control subject (**A**). The enlarged vestibular aqueduct is identified in a patient with EVA (**B**).

Profound hearing loss affects about 1 in 300 to 1 in 1000 newborns [1-4], and about one-half of these cases can be attributed to genetic factors [5]. About 51% of these cases are due to single nucleotide polymorphisms [5]. As to inheritance pattern among monogenic probands, about 1% is X-linked, 22% is autosomal dominant, and 77% is autosomal recessive [5]. Pendred syndrome (PS) is an autosomal recessive disorder characterized by congenital sensorineural hearing loss and goiter [6]. The causative gene for PS and EVA was identified to be *SLC26A4*[7,8]. Enlarged vestibular aqueduct (EVA) is a common inner ear malformation that can be diagnosed radiographically in patients with impaired hearing (Figure 1). EVA is frequently associated with PS [9-11]. In addition to PS, *SLC26A4* mutations also cause nonsyndromic hearing loss with EVA in the absence of a thyroid phenotype [12,13].

Previous studies revealed that the spectrum of *SLC26A4* mutations varied on the basis of ethnic background [14,15]. Tsukamoto et al. [15] demonstrated that *SLC26A4* mutations occurred in 90% of families with a history of PS and in 78% of families with a history of EVA in Japan. Among these *SLC26A4* mutations, H723R was suggested to have a founder effect in the Japanese population.

**Figure 2:**
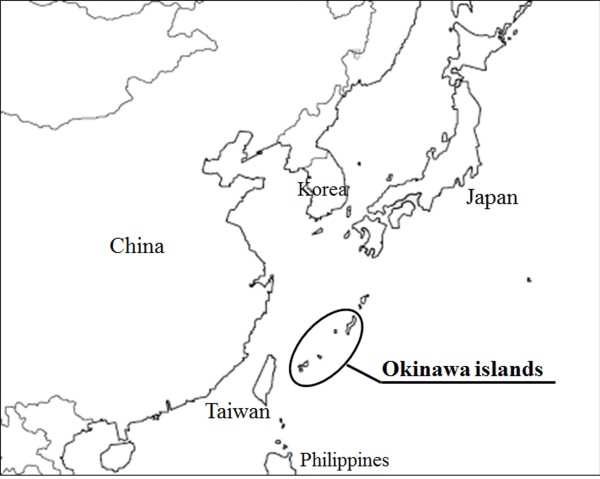
**Location of the Okinawa islands in relation to East Asia.** The Okinawa islands are located between Taiwan and the Japanese island of Kyushu. The Japanese archipelago comprises Hokkaido, Honshu, Kyusyu, and the Okinawa islands, as well as some smaller islands.

The Okinawa Islands are the southwestern-most islands of the Japanese archipelago (Figure 2). Previous studies suggested that there were substantial ancestral differences between Okinawa Islands the main islands of Japan [16]. In this study, we examined patients with EVA or PS from the Okinawa Islands to determine the frequencies and the genotypes of *SLC26A4* mutations and their clinical manifestations.

## Methods

### Subjects

From May 2008 to July 2012, 22 patients (8 males, 14 females; age range: 0–33 years; mean age: 5.8 years; median age: 8.5 years; Table 1) were diagnosed with PS or EVA in the Department of Otorhinolaryngology, Head and Neck Surgery of the University of the Ryukyus, Japan.

**Table 1:** Summary of clinical features of 22 patients

**Age (years)**			**CT**			**PTA**		**Vertigo**	**Thyroid**	
			**EVA**	**MD**	**VE**	**HL (dB)**	**Conductive hearing loss**		**Goiter**	**Thyroid function**
1	3	R	+	+	+	SO	unknown	-	-	normal
		L	+	+	+	SO	unknown			
2	14	R	+	+	-	105	+	-	+	normal
		L	+	+	-	96	+			
3	21	R	+	+	+	73	+	+	+	normal
		L	+	+	+	91	+			
4	21	R	+	-	-	81	+	+	+	normal
		L	+	-	-	85	+			
5	28	R	+	+	+	96	+	+	+	normal
		L	+	+	+	SO	+			
6	33	R	+	+	-	101	+	+	+	normal
		L	+	+	+	106	+			
7	1	R	+	+	-	SO	unknown	-	-	normal
		L	+	+	+	SO	unknown			
8	1	R	+	-	-	SO	unknown	-	-	normal
		L	+	-	-	103	unknown			
9	2	R	+	+	-	101	unknown	-	-	normal
		L	+	+	-	100	unknown			
10	12	R	+	-	-	95	+	-	+	normal
		L	+	-	-	100	+			
11	29	R	+	+	+	85	+	-	-	
		L	+	+	+	110	+			
12	0	R	+	-	-	55	unknown	+	-	normal
		L	+	-	-	73	unknown			
13	3	R	+	-	+	85	unknown	+	-	normal
		L	+	+	+	58	+			
14	5	R	+	+	+	95	+	+	-	normal
		L	+	+	+	93	+			
15	5	R	+	+	+	103	+	-	-	normal
		L	+	+	+	100	unknown			
16	6	R	+	-	-	81	+	+	-	normal
		L	+	-	-	91	+			
17	7	R	+	-	-	83	+	-	-	normal
		L	+	-	+	81	+			
18	14	R	+	+	+	96	+	-	+	normal
		L	+	+	+	91	+			
19	16	R	+	-	+	91	+	-	+	normal
		L	-	-	+	21	-			
20	26	R	+	-	-	98	+	+	-	normal
		L	+	-	+	103	+			
21	5	R	+	+	+	85	+	-	-	normal
		L	+	+	-	97	+			
22	10	R	+	-	-	53	+	-	-	normal
		L	-	-	-	15	-			

*EVA* enlarged vestibular aqueduct, *MD* Mondini malformation, *VE* vestibular enlargement, *PTA* pure tone audiogram, *HL* hearing level, *SO* scale out, *NA* no available data.

Prior to enrollment, all subjects provided a written informed consent. Our research protocol was approved by the Ethical Review Board of the University of the Ryukyus.

### Clinical manifestations of PS and EVA

Clinical history of 22 patients with neuro-otologic symptoms was recorded. A physical examination, including otoscopy, hearing level test, computed tomography (CT) scan of the temporal bones, and examination for thyroid goiter was conducted.

Depending on a subject’s ability, hearing level was determined using auditory brainstem response, conditioned orientated response, or pure tone audiogram. Hearing level was defined as the average of the hearing threshold at 0.5, 1.0, 2.0, and 4.0 kHz. Hearing was described as: normal, < 20 dB; mild impairment, 21–40 dB; moderate impairment, 41–70 dB; severe impairment, 71–90 dB; and profound impairment, >91 dB.

Neck palpation or echography of the neck was performed in all patients, to determine thyroid goiter. In addition, their serum levels of thyroid-stimulating hormone (TSH) and free thyroxine (FT4) were measured to evaluate thyroid function (normal values: 0.9–1.6 ng/dl and 0.5–5.0 mU/l, respectively). A perchlorate test was not performed.

High-resolution temporal bone CT was performed in all patients to determine if there were any other inner ear malformations in addition to EVA. EVA was defined as a vestibular aqueduct with a diameter of >1.5 mm at the midpoint between the common crus of the semicircular canal and the external aperture of the vestibular aqueduct on CT [17].

Mondini dysplasia was defined when the cochlea consisted of 1.5 turns in which the middle and apical turns had coalesced to form a cystic apex due to the absence of the interscalar septum [18,19].

Vestibular enlargement was defined when the ratio of the membranous vestibule diameter to the inner ear diameter of the lateral semicircular canal was >1.2 [20].

Vertigo was investigated based on spontaneous nystagmus, caloric vestibular test or patients’ self-reporting of past episode. The spontaneous nystagmus was evaluated using Frenzel’s glass or infrared CCD camera (IRN-1, Morita, Kyoto, Japan).

### *SLC26A4* genotyping

Genomic DNA was extracted from whole blood using a QIAamp DNA Blood Mini Kit (Qiagen, Hilden, Germany). To detect mutations, all 21 exons and the exon–intron junctions of *SLC26A4* were sequenced for all subjects. A 35 step cycle of Polymerase chain reactions (PCR) was performed as follows: initial denaturation at 94°C for 5 min; 35 cycles of 94°C for 40 s, 60°C for 40 s, and 72°C for 1 min; and a final extension at 72°C for 5 min. PCR reactions were run using a programmable thermal cycler (Verti™ 96-Well Thermal Cycler, Applied Biosystems, CA, USA).

PCR products were purified using a Wizard® SV Gel and PCR Clean-Up System (Promega, WI, USA) and directly sequenced using an ABI PRISM 3130 × l Genetic Analyzer (Applied Biosystems). The sequences obtained were aligned and compared using the BLAST program with known human genome sequences available in the GenBank database.

We surveyed the substitution IVS15 + 5G > A in 100 healthy objects as control.

The genotype of the IVS15 + 5G > A was detected by digestion of the PCR product with the restriction enzyme SspI (New England Biolabs, Ipswich, MA, U.S.A).

### Total RNA isolation and reverse-transcription

Total RNA was isolated from leukocytes using a QIAamp RNA Blood Mini Kit (Qiagen) according to the manufacturer’s protocol. Before cDNA synthesis, residual DNA was removed by incubation with RNase-free DNase I (Ambion Inc., City, TX, USA). Then, total RNA was reverse transcribed using a TaKaRa Prime Script High Fidelity RT® Kit (TaKaRa, Tokyo, Japan) according to the manufacturer’s protocol. Possible contaminating genomic DNA in RNA samples was determined by electrophoresis.

### Quantitative nested real-time PCR

Nested real-time quantitative (q) PCR was performed to investigate the level of *SLC26A4* expression in the blood.

### First-step PCR (conventional PCR)

A conventional PCR assay was performed in a 10 μl reaction mixture that included 2 μl of cDNA, 0.5 units of DNA Taq polymerase (TaKaRa), 2.5 mM deoxynucleotide triphosphates (dNTPs), 1 μM forward and reverse primers for first-step PCR (Table 2), 10 × buffer, and 1.875 mM MgCl_2_, with distilled water (H_2_O) for the final reaction volume of 10 μl. A 33 step cycle of PCR were performed as follows: 94°C for 5 min, 33 cycles of 94°C for 30 s, 60°C for 30 s, 72°C for 40 s, and a final extension at 72°C for 5 min.

**Table 2:** Primer sequences used for nested real-time PCR

**Nested PCR assay**			**Sequence**	**PCR product size (bp)**
First-step PCR (external primer)	Exon 14	forward	TCTTGGAATGGCCTTGGAAGC	282
	Exon 17	reverse	TGAAACAGCATCACTTATGATGC	
Second-step PCR (internal primer)	Exon 15	forward	TGAAGAACCTCAAGGAGTGAAG	154
	Exon 16	reverse	TTTCTGTATTTTCCTCAGCGCT	

### Second-step PCR (quantitative nested PCR)

Following the first PCR, a second PCR was performed using a set of internal primers (Table 2). The reaction mixture contained 1 μl of the first PCR product (diluted 10-fold), 10 μl of SYBR Premix Ex Taq, and 0.2 μM of the internal forward and reverse primers; the final reaction volume was adjusted to 20 μl with distilled H_2_O. A Light Cycler real-time quantitative PCR system (Roche, Basel Switzerland) was used for amplification and detection of the PCR products. A 40 step cycle of thermal cycler program was performed as follows: denaturation at 95°C for 5 min; 40 cycles of 95°C for 10 s, 60°C for 20 s, and 72°C for 40 s; followed by recording the fluorescence values after each elongation step and melting curve analysis with denaturation at 95°C for 5 s, annealing at 65°C for 1 min, and redenaturation by increasing the temperature to 95°C. The second-step PCR products were separated by 1.5% agarose gel electrophoresis, stained with ethidium bromide, and visualized by UV transillumination. For this analysis, we used three control subjects with no mutations (wild type), three patients compound heterozygous for IVS15 + 5G > A/H723R, and three patients homozygous for IVS15 + 5G > A.

### Validation of comparative CT (2^−ΔΔCT^) method and calculations for quantifying *SLC26A4* mRNA

We used the CT (2^−ΔΔCT^) method by assuming approximately equal amplification efficiencies for both target and reference genes. This prerequisite was verified by performing a validation experiment using both *SLC26A4* and a housekeeping gene. Calculations were made using the comparative CT (2^−ΔΔCT^) method. *GAPDH* (glyceraldehyde 3-phosphate dehydrogenase), *PGK-1* (phosphoglycerate kinase 1), and *ACTB* (actin beta) were used as internal reference genes for PCR normalization with regard to the amount of RNA added to the reverse transcription reactions. Normalized results were expressed as the mean ratio of *SLC26A4* mRNA to *GAPDH* mRNA, *PGK-1* mRNA, and *ACTB* mRNA. To evaluate relative transcript levels, the threshold cycle value (Ct) of each sample was used to calculate and compare the ΔCt of each sample to that of the control subject and patients with a compound heterozygous for IVS15 + 5G > A/H723R, and a homozygous for IVS15 + 5G > A. ΔΔCT was also calculated to compare the transcript levels in the control subject, and patients with a compound heterozygous for IVS15 + 5G > A/H723R, and a homozygous for IVS15 + 5G > A. The transcript levels were calculated in each genotype with three subjects and each subject was calculated in triplicate.

## Results

### Mutation analysis for *SLC26A4*

**Figure 3:**
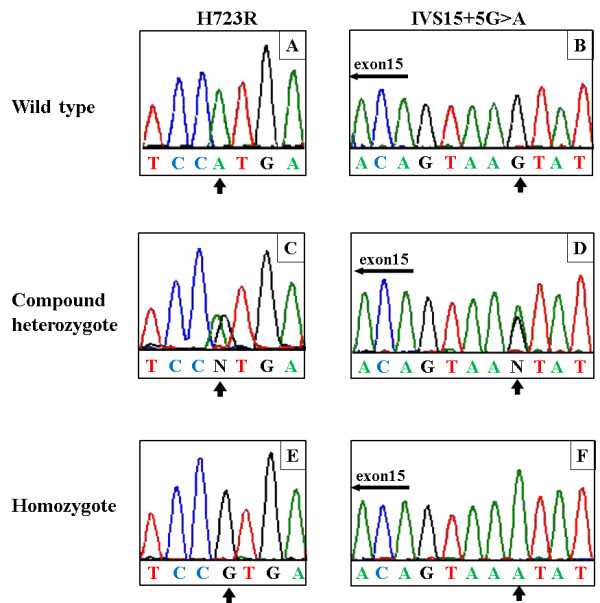
**Examples of direct sequence analysis of the *SLC26A4 *gene.** Representative results of H723R and the IVS15 + 5G > A mutation analysis are shown. Genomic sequences of the *SLC26A4* gene in normal individuals (**A**), (**B**). A compound heterozygous mutation for IVS15 + 5G > A/H723R (**C**), (**D**). A homozygous mutation for H723R (**E**). A homozygous substitution of IVS15 + 5G > A (**F**). The arrows indicate the variant nucleotide.

By direct DNA sequence analysis, *SLC26A4* mutations were observed in 21 of 22 patients. Among the 21 patients with mutations, a compound heterozygous mutation for IVS15 + 5G > A/H723R was identified in nine patients (Figure 3C, D), a homozygous mutation for H723R was identified in five patients (Figure 3E), and a homozygous substitution of IVS15 + 5G > A was identified in six patients (Figure 3F). A compound heterozygous substitutions for IVS15 + 5G > A/T527P was identified in one subject. We could not identify any *SLC26A4* mutations in one subject (Table 3). We could not find the substitution IVS15 + 5G > A in 100 control objects.

**Table 3:** **Distribution of *SLC26A4 *genotypes of 22 patients**

	**Age at onset of hearing loss (years)**	**Age at genetic test (years)**	**Sex**	**Allele 1**	**Allele 2**
1	0	3	M	IVS15 + 5G > A	IVS15 + 5G > A
2	2	14	F	IVS15 + 5G > A	IVS15 + 5G > A
3	3	21	F	IVS15 + 5G > A	IVS15 + 5G > A
4	2	22	F	IVS15 + 5G > A	IVS15 + 5G > A
5	0	23	M	IVS15 + 5G > A	IVS15 + 5G > A
6	0	29	F	IVS15 + 5G > A	IVS15 + 5G > A
7	0	1	F	H723R	H723R
8	1	1	F	H723R	H723R
9	4	2	M	H723R	H723R
10	0	12	F	H723R	H723R
11	5	29	M	H723R	H723R
12	0	0	M	IVS15 + 5G > A	H723R
13	2	3	M	IVS15 + 5G > A	H723R
14	0	5	F	IVS15 + 5G > A	H723R
15	1	5	F	IVS15 + 5G > A	H723R
16	0	6	F	IVS15 + 5G > A	H723R
17	2	7	F	IVS15 + 5G > A	H723R
18	2	14	F	IVS15 + 5G > A	H723R
19	7	16	F	IVS15 + 5G > A	H723R
20	5	26	M	IVS15 + 5G > A	H723R
21	1	5	M	H723R	T527P
22	7	10	F	ND	ND

*ND* not determined.

### Clinical characteristics

Table 1 summarizes the clinical characteristics of all 22 subjects. High-resolution temporal bone CT scans revealed that bilateral EVA was present in 20 patients and unilateral EVA was present in other two. Mondini dysplasia and vestibular enlargement was observed in 17 ears (17/44; 39%) and 22 ears (22/44; 50%), respectively.

Hearing loss grades in the affected ears ranged from moderate to profound in the patients with EVA (Table 1). The hearing levels of the two unaffected ears were normal and mild hearing loss, respectively. Table 4 shows the hearing level distributions based on genotypes. No significant differences were expected in the distributions for hearing level among the five genotype groups due to the small sample of only 22 patients.

**Table 4:** Clinical features in different genotype groups

**Genotype**	**Hearing level**					**CT**		**Vertigo**
	**Normal**	**Mild**	**Moderate**	**Severe**	**Profound**	**MD**	**VE**	
IVS15 + 5 G > A homozygous (n = 6)	0	0	0	3	9	6/12	6/12	4/6
H723R homozygous (n = 5)	0	0	0	1	9	4/10	3/10	0/5
IVS15 + 5 G > A/H723R (n = 9)	0	1	2	4	11	5/18	11/18	4/9
IVS15 + 5G > /T527P (n = 1)	0	0	0	1	1	2/2	1/2	0/1
No mutation (n = 1)	1	0	1	0	0	0/2	0/2	0/1
Subtotal	1	1	3	9	30	17/44	21/44	8/22
Total			44					

Normal: ≤20 dB; Mild: 21–40 dB; Moderate: 41–70 dB; Severe: 71–90 dB; Profound: >91 dB.

*MD* Mondini malformation, *VE* Vestibular enlargement, *CT* computed tomography.

Neck examinations revealed thyroid goiters in 8 of 22 patients. Overall, 0% (0/11) and 73% (8/11) of the patients younger and older than 10 years of age, respectively, had a thyroid goiter. Their serum FT4 and TSH levels were within the normal ranges. There is no relation between occurrence of goiter and mutation genotypes.

### *SLC26A4* expression in patients with IVS15 + 5G > A

**Figure 4:**
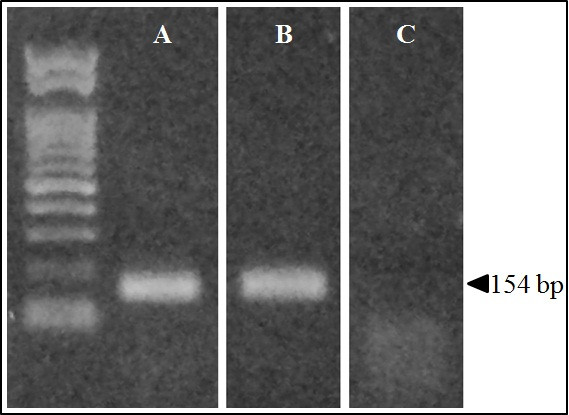
**Expression of the *SLC26A4 *gene in patients with PS or EVA.** The expected RT-nested PCR amplification product of *SLC26A4* was 154 base pairs (bp) in length. Agarose gel electrophoresis shows the 154 bp band for the control subject (**A**) and the patient with IVS15 + 5G > A/H723R compound heterozygous mutation (**B**); however, there was no band for the patient with IVS15 + 5G > A homozygous substitution (**C**).

Electrophoretic separation of the real-time PCR products did not exhibit any bands in patients with the homozygous substitution for IVS15 + 5G > A (Figure 4C).

**Figure 5:**
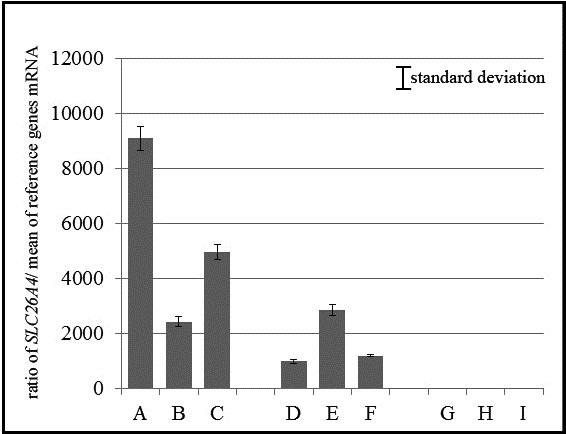
**Relative expression of the *SLC26A4 *gene in control subjects and in patients with a homozygous mutation of IVS15 + 5G > A or compound heterozygous mutation of IVS15 + 5G > A/H723R.** The ratio of SLC26A4 mRNA to GAPDH mRNA is shown in three control subjects (**A**, **B**, **C**), three patients with compound heterozygous mutation of IVS15 + 5G > A/H723R (**D**, **E**, **F**), and three patients with IVS15 + 5G > A homozygous substitution (**G**, **H**, **I**). No expression of *SLC26A4* was observed in the three patients with the IVS15 + 5G > A homozygous substitution (**G**, **H**, **I**). All experiments were done in tripricate.

Because the *SLC26A4* expression levels were not high in blood samples, we investigated its expression using nested real-time qPCR for three control subjects, three patients with the compound heterozygous mutation for IVS15 + 5G > A/H723R, and three patients with the homozygous substitution for IVS15 + 5G > A. The control subjects had normal hearing without any malformations of the inner or middle ear and no family history of hearing loss. After obtaining a written informed consent, blood samples were collected from each subject and were subjected to Real-time PCR with SYBR Green and the expression level was evaluated using the comparative CT (2^-ΔΔCT^) method. The relative *SLC26A4* expression levels in the control no.1, control no.2 and control no.3 with no *SLC26A4* mutations were 9089 ± 441.5 (standard deviation), 2417 ± 189.5, and 4956 ± 260.4 respectively. In patient no.12, patient no.14 and patient no.16 with a compound heterozygous mutation for IVS15 + 5G > A/H723R were 979.5 ± 79.12, 2846 ± 206.5 and 1183 ± 33.93 respectively. In patient no.1, patient no.2 and patient no.4 with a homozygous substitution for IVS15 + 5G > A were 1.96 × 10^-4^ ± 7.66 × 10^-5^, 5.76 × 10^-5^ ± 3.37 × 10^-6^ and 4.35 × 10^-5^ ± 8.09 × 10^-6^ respectively (Figure 5).

Based on the results of both electrophoresis and RT-nested qPCR, no *SLC26A4* expression was observed in patients with homozygous substitution of IVS15 + 5G > A.

## Discussion

### Correlations between *SLC26A4* genotypes and hearing phenotypes

Hearing loss in patients with EVA and PS is usually apparent at the pre- or perilingual stage [6,21]. Hearing loss in EVA and PS is sensorineural with some mixed hearing loss in the low-frequency range [22-27]. The hearing level sometimes deteriorates suddenly and may be followed by a partial recovery, such as with fluctuating hearing loss [28,29]. In our study, hearing loss was detected at the pre- or perilingual stage in all cases except for two cases of unilateral EVA. However, in all cases, hearing levels eventually deteriorated to severe or profound loss (Table 1) and were permanent with or without hearing fluctuation or stepwise hearing deterioration. No significant differences were observed in the hearing levels among the five genotypes (Table 4).

### Correlations between *SLC26A4* genotypes and thyroid phenotype

*SLC26A4* encodes for the 86 kDa transmembrane protein pendrin [7,30]. In the thyroid, this protein acts as co-transporter of chloride and iodine in the thyroid [31,32]. In PS patients, a mutation in *SLC26A4* results in reduced pendrin-induced chloride and iodide transport and, ultimately, goiter [33].

Goiter usually develops around the end of the first decade of life or during young adulthood, although the time of onset and severity vary considerably among patients [12,34], and even within families [35]. Despite an impaired incorporation of iodide, most patients with PS are clinically and biochemically euthyroid [21,34,36].

To our knowledge, no previous studies have investigated correlations between *SLC26A4* genotypes and the thyroid phenotype. In the present study, PS was diagnosed in 8 of 11 patients older than 10 years of age, but not in any of the 11 patients who were younger than 10 years of age. This indicates that it is difficult to diagnose PS before the age of 10 years.

Thyroid function was normal in all of the 21 patients we examined, as demonstrated by their normal serum concentrations of FT4 and TSH. There were no significant differences in serologic thyroid test results and goiter status among patients with homozygous substitution for IVS15 + 5G > A, the H723R homozygous mutation, or compound heterozygous mutation for IVS15 + 5G > A/H723R. Therefore, our results indicate that serologic testing of FT4 and TSH levels is not useful to distinguish between individuals with PS or EVA.

### Distributions of *SLC26A4* mutations in EVA and PS patients in Okinawa Islands

It was previously reported that the spectrum of *SLC26A4* mutations varied based on ethnic background [35,36]. H723R and IVS7-2A > G are prevalent alleles that account for the majority of the observed *SLC26A4* mutations in East Asian populations [35]. In the Japanese population, H723R was the most common mutation [15,36,37]. In Chinese and Taiwanese populations, IVS7-2A > G was the most common mutation [38-40], whereas in the Korean population, H723R and IVS7-2A > G were the most frequent and accounted for 60.2% (47/78) and 30.7% (24/78) of the mutated alleles, respectively [41].

Ancestral differences have been reported between people from Okinawa Islands and those from the main islands of Japan based on single-nucleotide polymorphism genotypes [16]. We analyzed *SLC26A4* mutations among 22 patients with EVA or PS from 21 unrelated families. H723R have been reported as the most common mutation found in the main islands of Japan. As with H723R mutation, IVS15 + 5G > A substitution was also identified most frequently in 15 of 22 of our Okinawa patients. The substitution of IVS15 + 5G > A in one allele have been reported only 10 cases in Asian populations [36,42-45]. Thus, IVS15 + 5G > A was the characteristic *SLC26A4* gene mutation among patients in Okinawa Islands, indicating a difference in the spectrum of *SLC26A4* mutations among patients in Okinawa Islands compared with patients in other populations. These results suggest that this *SLC26A4* mutation may have originated from a common ancestor.

### Pathogenic effect of IVS15 + 5G > A substitution

The heterozygous substitution of IVS15 + 5G > A has been assumed to cause aberrant splicing [36,42-45]. However, Yang et al. [42] could not find any abnormal RT-PCR products related to the size for SLC26A4 sequence analysis in patients with splice mutation. Because its pathogenicity was only implicated on the basis of uncommon polymorphisms, the pathogenic potential of IVS15 + 5G > A still remains unknown.

Substitutions near the canonical splice sites are difficult to classify as pathogenic or non-disease causing. Because such substitutions affect proper RNA splicing but some substitutions do not cause any effect [46-48]. Thus, it is important to determine the pathogenic effect of a particular substitution near the donor site by mRNA analysis [48]. We investigated *SLC26A4* expression in patients with compound heterozygous mutation for IVS15 + 5G > A/H723R and homozygous substitution for IVS15 + 5G > A by RT-PCR and RT-real time PCR by targeting genes around these mutations. No aberrant PCR products were detected in the patient with heterozygous substitution of IVS15 + 5G > A (Figure 4B), which suggests that IVS15 + 5G > A does not cause aberrant splicing, as also argued by Yang et al. However, in patients with the homozygous substitution of IVS15 + 5G > A, *SLC26A4* was not expressed, as shown in Figure 4. In addition, for patients with the heterozygous substitution, *SLC26A4* expression was reduced from the normal control level. These findings suggest that IVS15 + 5G > A disrupts pre-mRNA splicing and causes the loss of *SLC26A4* expression. The patients in Yang et al. [42] were heterozyote so that Yang et al. [42] most likely amplified the non-mutated allele. Taken together, our results indicate that the substitution of IVS15 + 5G > A is a loss-of-function mutation caused by a loss of *SLC26A4* expression.

## Conclusions

We found no correlations between the type of *SLC26A4* mutation and hearing levels or the thyroid phenotype. Moreover, thyroid testing using serum FT4 and TSH levels was not useful for distinguishing between individuals with PS and EVA.

The substitution of IVS15 + 5G > A in the *SLC26A4* was unique and the most common in PS and EVA patients from Okinawa Islands. This supports that the spectrum of *SLC26A4* mutations differs by geographic area in East Asia. Our qPCR results for *SLC26A4* indicate that the substitution of IVS15 + 5G > A should be a pathogenic mutation that leads to a loss of *SLC26A4* expression and results in a phenotype of PS and EVA.

## Competing interests

The authors declare that they have no competing interests.

## Authors’ contributions

AG diagnosed the patients, collected clinical data, performed the experiments, and wrote the manuscript. TK, KY, and SU carried out data analysis. KN, TT, and MS edited the manuscript and supervised the project. All authors read and approved the final manuscript.

## Pre-publication history

The pre-publication history for this paper can be accessed here:

http://www.biomedcentral.com/1471-2350/14/56/prepub
